# Preschool wheezing

**DOI:** 10.1186/1824-7288-41-S2-A64

**Published:** 2015-09-30

**Authors:** Francesca Santamaria

**Affiliations:** 1 Department of Translational Medical Sciences, Section of Pediatrics, Federico II University, Naples, Italy

## 

Pediatricians face many challenges when diagnosing wheezing in preschool children. These diagnostic challenges are compounded by variations in the natural history of early stage asthma, which are not fully understood, since early childhood wheezing and asthma are heterogeneous disorders with many phenotypic and variable expressions. Several risk factors related to genetic, prenatal, and postnatal environment are associated with preschool wheezing. Findings from cohort studies have shown that preschool children with wheeze have deficits in lung function at 6 years of age that persisted until early and middle adulthood, suggesting increased susceptibility in the first years of life that might lead to persistent sequelae [1].

Since no standard definition for the type, severity, or frequency of symptoms exist for this age group, clear evidence-based recommendations are lacking. Without adequate guidance, pediatricians are left to make diagnostic and treatment decisions, which can lead to undertreatment of asthmatics and overtreatment of transient wheezers. New guidelines and/or Consensus documents that specifically address the challenges of diagnosing asthma in this particular age group have recently been published, and researchers are actively seeking new methods and techniques through epidemiological studies to assist primary care clinicians in the diagnostic process [2-5].

Treatment of young children with asthma remains poorly defined and very controversial. A study has described the prescribing patterns among primary care physicians in Italy, and concluded that child characteristics alone are not sufficient to explain how physicians decide to prescribe maintenance treatment and which specific therapy to assign [6]. Daily inhaled corticosteroids seem to be the most effective therapy for recurrent wheezing in trials of children with interim symptoms or atopy. Intermittent high-dose inhaled corticosteroids are effective in moderate-to-severe viral-induced wheezing without interim symptoms. However, the role of corticosteroids in treating acute asthma in young children has been questioned, is currently being carefully evaluated, and requires evidence-based directions [7-9]. The role of leukotriene receptor antagonist is less clear, and should hopefully be evaluated further in larger study groups [10]. Interventions to modify the short-term and long-term outcomes of preschool wheeze should be a research priority.
